# Autoantibodies to NR2A Peptide of the Glutamate/NMDA Receptor in Patients with Seizure Disorders in Neuropsychiatric Systemic Lupus Erythematosus

**DOI:** 10.1155/2017/5047898

**Published:** 2017-01-05

**Authors:** Yan Yang, Chao Yuan, Shu-qun Shen, Xue-er Wang, Qing-hua Mei, Wen-qing Jiang, Qin Huang

**Affiliations:** ^1^Guangdong No. 2 Provincial People's Hospital, Courtyard No. 466, Middle Xingang Road, Haizhu District, Guangzhou 510317, China; ^2^Department of Neurology, Nanfang Hospital, Southern Medical University, No. 1838 North Guangzhou Avenue, Guangzhou 510515, China; ^3^TEDA International Cardiovascular Hospital, Medical College, Nankai University, Tianjin, China; ^4^School of Public Health, Southern Medical University, No. 1023 South Shatai Rd., Guangzhou, Guangdong 510515, China; ^5^Department of Histology and Embryology, School of Basic Medical Sciences, Southern Medical University, Guangzhou 510515, China; ^6^Nanfang Hospital, Southern Medical University, Guangzhou 510515, China; ^7^Department of Rheumatology, Nanfang Hospital, Southern Medical University, No. 1838 North Guangzhou Avenue, Guangzhou 510515, China

## Abstract

*Objective*. Seizure disorders are one of the most disabling, life-threatening, and the least understood syndromes associated with neuropsychiatric SLE (NPSLE). N-Methyl-D-aspartate (NMDA) receptors are a subgroup of the glutamate receptor family, whose NR2A subunit was found on neuronal cells (anti-NR2A) in NPSLE patients with different types of epilepsy. The present study was conducted to determine the serum levels of anti-NR2A antibodies in a large group of SLE patients, to investigate the possible correlation between the presence of the NR2A specific antibodies and NPSLE-related seizure disorders.* Methods and Results*. The study population consisted of 107 SLE patients and 43 age- and sex-matched healthy controls. 73 SLE patients had active disease. 36 of these had NPSLE. NMDA levels were measured by ELISA. Clinical and serological parameters were assessed according to routine procedures. The levels of anti-NR2A antibodies were significantly higher in NPSLE patients, compared with non-NPSLE patients and healthy controls. Furthermore, the levels of NPSLE in patients with seizure disorders were shown to be higher than in those with cognitive dysfunction and other CNS symptoms, however, without significance. Increase in serum anti-NR2A antibodies levels correlated to anti-dsDNA antibody and SLEDAI as well as complement levels.* Conclusion*. We suggest that anti-NR2A antibodies play a role in the pathogenesis of NPSLE with seizure disorders.

## 1. Introduction

Systemic lupus erythematosus (SLE) is a systemic autoimmune disease characterized by multiorgan involvement and numerous immunological abnormalities [1]. Approximately 10–80% of patients with SLE suffer from neuropsychiatric disorders [2, 3]. Seizure disorders are one of the most disabling, life-threatening, and the least understood of the 19 syndromes associated with neuropsychiatric SLE (NPSLE), classified by the American College of Rheumatology (ACR) [4].

Several studies have shown an apparent correlation between NPSLE and autoantibodies. For example, antiphospholipid antibodies, anti-ribosomal P antibodies, and N-methyl-D-aspartate (NMDA) receptor antibodies are associated with strokes and other central nervous system (CNS) symptoms. NMDA receptors are ligand-gated ion channels with important roles in synaptic transmission and CNS plasticity, which is necessary for cognition, memory, and neuronal survival. Excessive NMDA receptor activation is implicated in multiple brain disorders, including stroke, chronic neurodegeneration, epilepsy, and schizophrenia [5–10].

Of note, a recent study has found antibodies reactive with NMDA receptor NR2A subunit on neuronal cells (anti-NR2A) in ~20% of NPSLE patients with different types of epilepsy [11]. However, neither the precise mechanism of the elevation of anti-NR2A nor its relevance with the severity of seizure disorders in NPSLE is understood. Additionally, the comparison between serum anti-NR2A antibody in seizure disorders and other CNS symptoms such as cognitive dysfunction has not been investigated in detail.

The present study was conducted to determine the serum levels of anti-NR2A antibodies in a large group of SLE patients, to investigate the possible correlation between the presence of the NR2A specific antibodies and NPSLE-related seizure disorders.

## 2. Patients and Methods

### 2.1. Patients

This retrospective study included patients admitted to our hospital from January 2011 to January 2015. 107 adults with SLE were included in the study, and 43 age- and sex-matched healthy people were enrolled as control group. The diagnostic criteria for SLE were based on the 1997 revised American College of Rheumatology (ACR) classification [12]. Exclusion criteria were severe renal failure, severe infections, any mental disability that precluded performing the psychiatric evaluation, history of severe mental illness, estimated premorbid intelligence quotient of <80, and glucocorticoid-induced mania. The control individuals demonstrated no major underlying diseases and did not take any medicines including antioxidant agents.

Disease activity was recorded according to SLE disease activity index 2000 (SLEDAI-2k) [13]. Patients with SLEDAI ≥4 were considered active, and patients with a SLEDAI score <4 were considered to have quiescent disease. Of the 107 SLE patients, 34 were patients with quiescent disease visiting the outpatient clinic. The other 73 patients were selected for the presence of active disease. Characteristically, active SLE patients can be classified into two groups, which are NPSLE and non-NPSLE groups, according to the ACR classification [13]. Clinical data were obtained from all patients. Data acquisition included medical history, physical examination, and clinical symptoms. The types of seizures and epileptic syndromes were classified according to the guidelines of the International League against Epilepsy [14, 15]. Seizures were classified as generalized convulsive (e.g., tonic spasm, clonic, or tonic-clonic) or nonconvulsive (e.g., absence or myoclonic) or as partial (focal), depending on the clinical presentation [16].

The serologic variables related to SLE were collected from chart review. These included antinuclear antibody, anti-dsDNA antibody, SSA, SSB, anti-ribosomal P antibodies, and C3 and C4 levels. They were detected by the same testing kits (Euroimmun, Lübeck, Germany) at the same laboratory. Peripheral blood was collected for measurement of anti-NR2A antibody. All the serum samples were kept frozen at −80°C. The study was approved by the human ethics committee, and written informed consent was obtained from all participants.

### 2.2. Measurement of Anti-NR2A Antibody

Antibodies against the NR2A peptide were measured by ELISA using synthetic DWEYSVWLSN (DWEYS peptide) (BGI Genomics) as previously described [17–19]. Briefly, the 96-well microtiter plates (Nunc, Denmark) were coated with 0.025 *μ*g peptide per well at 4°C overnight. Plates were washed 4 times with phosphate-buffered saline containing 0.05% Tween-20 (PBS-T) (Sigma). The wells were blocked with Block Ace in PBS for 2 hours at 37°C. The wells were then washed with PBS-Tween, and plates were incubated with sera dilutions (1 : 50) for 3 hours at 37°C. Following further washing, peroxidase-conjugated goat anti-human IgG (BGI Genomics) was added at a 1 : 5000 dilution. Tetramethyl benzidine (Sigma) was added to each well and incubated at 37°C. After 15 min, the reaction was stopped by the addition of 2 N H_2_SO_4_. Optical density (OD) at 450 nm was measured by an ELISA reader (Bio-Tec, USA). The OD value of anti-NR2A antibody was estimated by subtracting the OD values for BSA-conjugated peptide and BSA alone. The intra- and interassay variances (coefficients of variation) for anti-NR2A were 5 and 15%, respectively.

### 2.3. Statistical Analysis

Statistical analyses were performed by using the chi-square test to compare frequencies, the *t*-test to compare mean values, and the Mann–Whitney *U* test to compare median values, using the statistical package GraphPad Prism, version 6.0b (GraphPad Software Inc., San Diego, CA, USA). Correlation coefficients were calculated as Pearson's correlation. A *P* value < 0.05 was considered significant.

## 3. Results

### 3.1. Clinical and Laboratory Characteristics of the Study Population

The clinical and laboratory characteristics of the study population were summarized in Tables 1–3. 92 female (86%) and 15 male (14%) patients were included in the SLE group; ages ranged from 18 to 62 years. The control group comprised 43 healthy volunteers, 32 women (74%), and 11 men (26%), aged 19 to 62 years (Table 1). 73 SLE patients had active disease, 36 of whom were involved with neuropsychiatric manifestations. In the active SLE group, the ages of those in the NPSLE subgroup were significantly lower than of those in the non-NPSLE subgroup (Table 2). Eighty percent of the patients were treated with steroids at the time of the study and 51% were treated with hydroxychloroquine. And there was no significant difference in medical history. The frequency of seizure was significantly higher in active disease group compared with the quiescent disease group (*P* = 0.002) and also higher in the NPSLE group than in the non-NPSLE group (*P* < 0.0001). In the NPSLE patients, consciousness disorders were significantly different between seizure group and group without seizure (*P* < 0.0001) (Table 3).

The levels of antinuclear antibody were similar between active disease group and quiescent disease group, but SLE disease activity indexes (SLEDAI) (*P* < 0.0001) and levels of C3 (*P* < 0.0001), C4 (*P* < 0.0001), and anti-dsDNA (*P* < 0.0001) were significantly higher in active disease group than in quiescent disease group. Furthermore, SLE disease activity indexes (SLEDAI) (*P* < 0.0001) and levels of ds-DNA (*P* < 0.0001) were significantly higher in NPSLE group than in the non-NPSLE group. Compared with patients without seizure in NPSLE group, SLEDAI scores in the seizure group were significantly higher.

**Figure 1:**
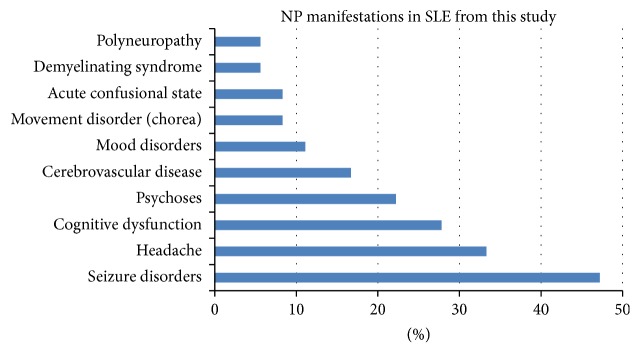
Distribution of NP manifestations in NPSLE patients.

The occurrence of NP disease was determined using the ACR case definitions for NPSLE, which includes a detailed glossary and diagnostic guidelines for 19 NP syndromes. Ten of the 19 ACR NPSLE syndromes were identified and 36 patients had a total of 69 NPSLE events. These can be segregated into central nervous system (CNS) and peripheral nervous system (PNS) subsets and into diffuse and focal NP subsets. CNS manifestations accounted for 94% (34/36 patients), while involvement of the PNS was 6% (2/36 patients). The majority of the manifestations were seizure disorders (*n* = 17; 47.2%), headache (*n* = 12; 33.3%), cognitive dysfunction (*n* = 10; 27.8%), and psychoses (*n* = 8; 22.2%). Other manifestations included cerebrovascular disease (six patients), mood disorders (four patients), movement disorder (chorea) (three patients), acute confusional state (three patients), demyelinating syndrome (two patients), and polyneuropathy (two patients). Data were shown in Figure 1.

### 3.2. Serum Anti-NR2A Antibody Levels in the Investigated Groups

**Figure 2:**
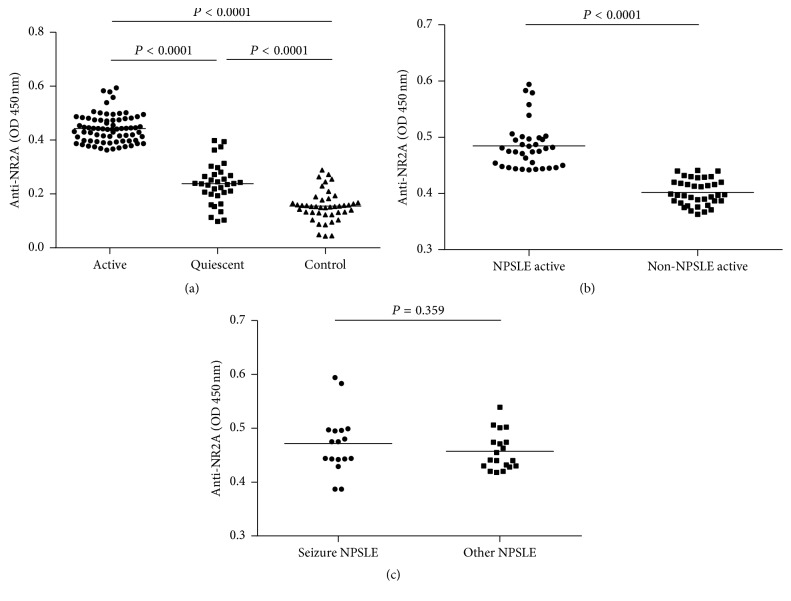
Anti-NR2A concentrations in serum from SLE patients and controls. (a) Serum anti-NR2A levels in SLE patients and controls using ELISA. Horizontal lines represent the median. (b) Comparison of serum anti-NR2A between NPSLE and non-NPSLE patients with ELISA. (c) Serum anti-NR2A measured by ELISA in NPSLE patients with seizure and those without seizure.

We first compared the serum anti-NR2A antibody levels of the controls with those of patients with SLE. In quiescent SLE patients, anti-NR2A levels significantly increased (0.238 (0.098 to 0.398)) compared to control group (0.155 (0.044 to 0.289)), while in patients with active disease anti-NR2A levels were even higher (0.432 (0.363 to 0.594)) (Figure 2(a)). Within the group of active patients, those with NP manifestations had higher anti-NR2A levels compared (0.464 (0.387 to 0.594)) to active patients with non-NP manifestations (0.402 (0.363 to 0.441)) (Figure 2(b)).

Subsequently, we compared serum anti-NR2A levels of the NPSLE patients with seizure and those without seizure. This comparison showed that anti-NR2A levels were similar in the two groups (0.471 (0.387 to 0.594) and 0.457 (0.418 to 0.539), resp.) (Figure 2(c)).

### 3.3. Correlations of Anti-NR2A Antibody Levels with Clinical and Serological Findings

**Figure 3:**
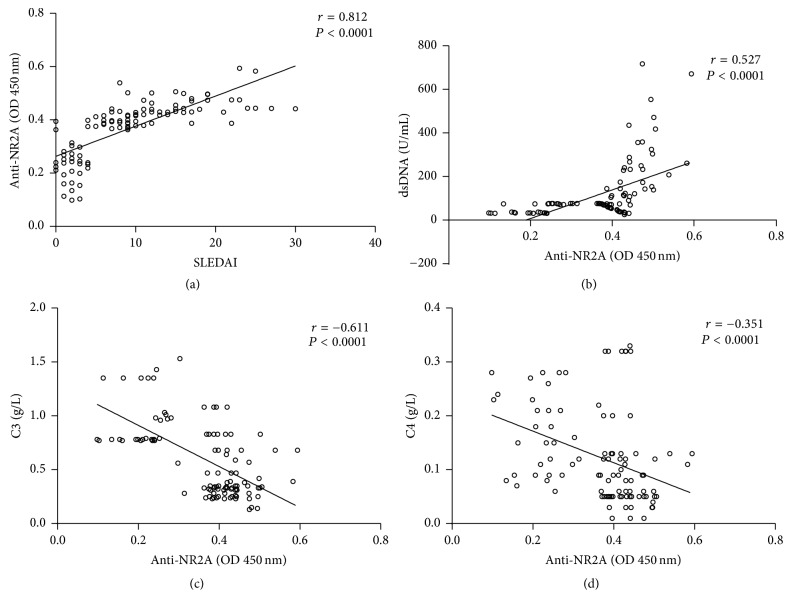
Correlations of anti-NR2A with anti-dsDNA antibodies, C3, C4, and SLEDAI in SLE patients.

As anti-NR2A antibody might be a marker of certain disease activity in SLE, we evaluated whether levels of anti-NR2A antibody were associated with clinical and serological parameters in SLE patients. We observed a correlation between anti-NR2A levels and SLEDAI (*P* < 0.0001, *r* = 0.812) (Figure 3(a)). Also, anti-NR2A levels showed a significant correlation with anti-dsDNA levels (*P* < 0.0001, *r* = 0.527) (Figure 3(b)). Complement proteins are involved in the pathogenesis of SLE and are considered biomarkers for disease activity. Therefore, we investigated the correlation of these factors with anti-NR2A. We observed a negative correlation in the total SLE group between C3, C4, and anti-NR2A levels (*P* < 0.0001, *r* = −0.611 and *P* < 0.0001, *r* = −0.351) (Figures 3(c) and 3(d)).

## 4. Discussion

We have demonstrated the association between anti-NR2A antibodies and various clinical manifestations and disease activity in SLE patients. Additionally, we compared the sera levels of anti-NR2A antibodies in NPSLE patients with seizure disorders and those with other CNS symptoms. Only one study has found anti-NR2A antibodies in ~20% of NPSLE patients with different types of epilepsy [9]. However, no specific correlation has been reported between anti-NR2A antibody and NPSLE in patients with seizure disorders. The main finding of the present study is that the levels of anti-NR2A antibodies were significantly higher in NPSLE patients, compared with non-NPSLE patients and healthy controls. Furthermore, the levels of NPSLE in patients with seizure disorders were shown to be higher than in those with cognitive dysfunction and other CNS symptoms, however, without significance.

NMDA receptors are a subgroup of the glutamate receptor family, responsible for mediating fast excitatory neurotransmission in CNS [20, 21]. It was reported that injection of anti-NR2 glutamate receptor binding antibodies (purified antibodies from the sera of SLE patients) into the mouse brain resulted in apoptosis of neuronal cells without signs of inflammation [18]. In chemical models, administration of agonists of the NMDA receptors induces convulsions in vivo by directly mediating epileptic depolarisation through the NMDA calcium channels [22–24]. In vitro studies in tissue resected from patients with epilepsy have associated NMDA receptor-mediated neurotransmission with seizure disorders [25–27]. In the present study, we found that the anti-NR2A antibodies existed in both SLE and healthy controls group in different levels. The levels were the highest in NPSLE patients with seizure disorders. Presumably, anti-NR2A antibodies were likely to participate in the physiological process, and their overexpression may play roles in epileptic seizure pathogenesis.

Many clinical manifestations of lupus appear to be mediated by autoantibodies, especially those for native DNA. The NR2A antibodies were originally reported to be anti-DNA antibodies. Degiorgio et al. demonstrate that a limited number of human SLE sera with dsDNA-specific antibodies cross-reacted with the NR2A and NR2B subunits of the NMDA receptors [18]. However, Husebye et al. reported that sera of 34 (31%) out of 109 SLE patients reacted specifically with the NR2A antibody, and no correlation was found between the presence of anti-NR2A and the presence of anti-dsDNA antibodies. Besides, they found no significant correlation between anti-NR2A antibody and NPSLE because of small numbers of patients with NPSLE (6, 5.5%) [11]. These findings are inconsistent with our observations; the number of patients with NPSLE was 36 (34%) in our study, and we found a significant correlation between anti-NR2A and anti-dsDNA antibodies.

Complement proteins (including C3 and C4) are previously used biomarkers for disease activity in SLE patients. We observed a significant negative correlation in the total SLE group between C3, C4, and anti-NR2A levels. The SLEDAI score was higher in patients with NPSLE, especially for seizure disorders, with a significant positive correlation with anti-NR2A levels. These findings were consistent and indicated that NPSLE patients have high-avidity anti-dsDNA reactive with anti-NR2A antibodies that activate complement strongly, leading to complement-dependent cytotoxicity.

NPSLE manifestations are demonstrated in almost 50% of SLE patients in most studies, which are classified into 19 syndromes by ACR, including diffuse disorders, such as cognitive dysfunction, mood disorders, and psychosis; focal disorders like seizures and strokes; and complex disorders, involving both diffuse and focal manifestations [2, 3, 28]. In the present study, seizure disorders, headache, and cognitive dysfunction were the most frequent of the NPSLE syndromes. Considering that headache is highly prevalent in the non-SLE population, we just compared the levels of anti-NR2A antibodies in NPSLE patients with seizure disorders and cognitive dysfunction. Results demonstrated that the two groups showed significantly higher anti-NR2A levels than the NPSLE patients with other NP syndromes. Furthermore, patients with seizure disorders expressed higher anti-NR2A antibodies than those with cognitive dysfunction, however, without significance. These findings were different from the previous studies [29–31].

NMDA is also influenced by various drugs. Corticosteroids generally decrease NMDA levels in the brain. Alfarez et al. have investigated the effects of corticosterone on NMDA expression in the rat hippocampus [32]. It appears that corticosteroid hormones suppress the NMDA expression at the mRNA and protein level in a subfield-specific way. However, no correlation has been reported between serum NMDA levels and corticosteroid levels. In our study, the patients were given complicated medications that might influence serum anti-NR2A levels such as immunosuppressive agents. However, we did not compare the medications and serum anti-NR2A antibodies. Further study controlling medications and serum anti-NR2A levels is needed to explore the hypothesis.

## 5. Conclusion

The present study demonstrates an increase in anti-NR2A antibodies levels in NPSLE patients, in particular in those with seizure disorders. Increase in serum anti-NR2A antibodies levels correlated to anti-dsDNA antibody and SLEDAI as well as complement levels. We suggest that anti-NR2A antibodies play a role in the pathogenesis of NPSLE with seizure disorders.

## Figures and Tables

**Table 1:** Comparison of clinical characteristics between quiescent and active SLE patients.

	SLE patients		Test value	*P* value
	Quiescent disease (*n* = 34)	Active disease (*n* = 73)		
Number of males/females	6/28	9/64	0.544	0.461
Age (years), median (range)	30 (21 to 62)	28 (18 to 62)	15.919	0.998
SLEDAI, median (range)	2 (0 to 4)	11 (5 to 30)	10.808	0.0001
Seizure, *n* (%)	0 (0%)	17 (23.3%)	9.413	0.002
ANA (U/mL), median (range)	175.0 (91.4 to 873.7)	166.6 (45.8 to 865.8)	−0.295	0.768
Anti-dsDNA (U/mL), median (range)	53.9 (31.6 to 76.5)	76.4 (25.6 to 717.5)	3.711	0.0001
Anti-ribosomal P antibodies, *n* (%)	8 (24%)	26 (36%)	1.563	0.211
SSA, *n* (%)	17 (50%)	38 (52%)	0.039	0.843
SSB, *n* (%)	8 (24%)	14 (19%)	0.269	0.604
C3 (g/L), median (range)	0.78 (0.28 to 1.53)	0.34 (0.13 to 1.08)	−8.134	0.0001
C4 (g/L), median (range)	0.16 (0.05 to 0.28)	0.06 (0.01 to 0.33)	−3.817	0.0001
Number with/without treatment	31/3	54/19		
Users of prednisone (%)	20 (59%)	42 (58%)	0.016	0.900
Dose (mg/day), median (range)	10 (2.5 to 50)	7.5 (2.5 to 100)		
Users of hydroxychloroquine (%)	20 (59%)	35 (48%)	1.099	0.295
Dose (mg/day), median (range)	400 (150 to 400)	400 (200 to 400)		

**Table 2:** Comparison of clinical characteristics between NPSLE and non-NPSLE in active SLE patients.

	Active SLE		Test value	*P* value
	NPSLE (*n* = 36)	Non-NPSLE (*n* = 37)		
Number of males/females	4/32	5/32	0.097	0.755
Age (years), median (range)	25 (18 to 60)	34 (19 to 62)	−2.973	0.004
SLEDAI, median (range)	17 (8 to 30)	9 (5 to 14)	9.174	0.0001
Seizure, *n* (%)	17 (47%)	0	22.776	0.0001
ANA (U/mL), median (range)	175.0 (45.8 to 696.9)	146.5 (66.6 to 873.7)	0.366	0.716
Anti-dsDNA (U/mL), median (range)	218.3 (21.6 to 747.5)	57.1 (31.6 to 115.3)	6.736	0.0001
Anti-ribosomal P antibodies, *n* (%)	14 (39%)	12 (32%)	0.332	0.565
SSA, *n* (%)	20 (56%)	18 (49%)	0.349	0.555
SSB, *n* (%)	6 (17%)	8 (22%)	0.289	0.591
C3 (g/L), median (range)	0.35 (0.13 to 1.53)	0.33 (0.23 to 1.08)	−0.732	0.467
C4 (g/L), median (range)	0.06 (0.01 to 0.33)	0.01 (0.01 to 0.33)	0.014	0.989
Number with/without treatment	32/4	26/11		
Users of prednisone (%)	19 (53%)	23 (62%)	0.658	0.417
Dose (mg/day), median (range)	15 (2.5 to 100)	10 (2.5 to 80)		
Users of hydroxychloroquine (%)	20 (56%)	21 (57%)	0.057	0.812
Dose (mg/day), median (range)	400 (150 to 400)	400 (200 to 400)		

**Table 3:** Comparison of clinical characteristics between patients with seizure and without seizure in NPSLE group.

	NPSLE		Test value	*P* value
	With seizure (*n* = 17)	Without seizure (*n* = 19)		
Number of males/females	2/15	2/17	0.014	0.906
Age (years), median (range)	25 (18 to 51)	24 (19 to 60)	−0.651	0.519
SLEDAI, median (range)	22 (16 to 30)	14 (8 to 19)	6.571	0.0001
Consciousness disorders, *n* (%)	12 (71%)	0	20.118	0.0001
ANA (U/mL), median (range)	175.0 (106.3 to 657.3)	175.0 (45.8 to 696.9)	0.499	0.621
Anti-dsDNA (U/mL), median (range)	233.0 (69.7 to 671.3)	207.8 (25.6 to 717.5)	−0.068	0.946
Anti-ribosomal P antibodies, *n* (%)	6 (35%)	8 (42%)	0.175	0.676
SSA, *n* (%)	11 (65%)	9 (47%)	1.092	0.296
SSB, *n* (%)	3 (18%)	3 (16%)	0.022	0.881
C3 (g/L), median (range)	0.32 (0.13 to 1.08)	0.38 (0.26 to 0.83)	−0.949	0.349
C4 (g/L), median (range)	0.05 (0.01 to 0.32)	0.09 (0.05 to 0.33)	−0.691	0.494
Number with/without treatment	13/4	14/5		
Users of prednisone (%)	11 (65%)	8 (42%)	1.839	0.175
Dose (mg/day), median (range)	7.5 (2.5 to 50)	10 (2.5 to 100)		
Users of hydroxychloroquine (%)	9 (53%)	8 (42%)	0.423	0.516
Dose (mg/day), median (range)	400 (150 to 400)	400 (200 to 400)		
